# Supplementary material for: Environmental circadian disruption re-writes liver circadian proteomes
Source: Nat Commun. 2024 Jul 1;15:5537. doi: 10.1038/s41467-024-49852-3 (PMC11220080; doi:10.1038/s41467-024-49852-3)
Supplement: Supplementary file 1 — Supplementary Information [file 41467_2024_49852_MOESM1_ESM.pdf]

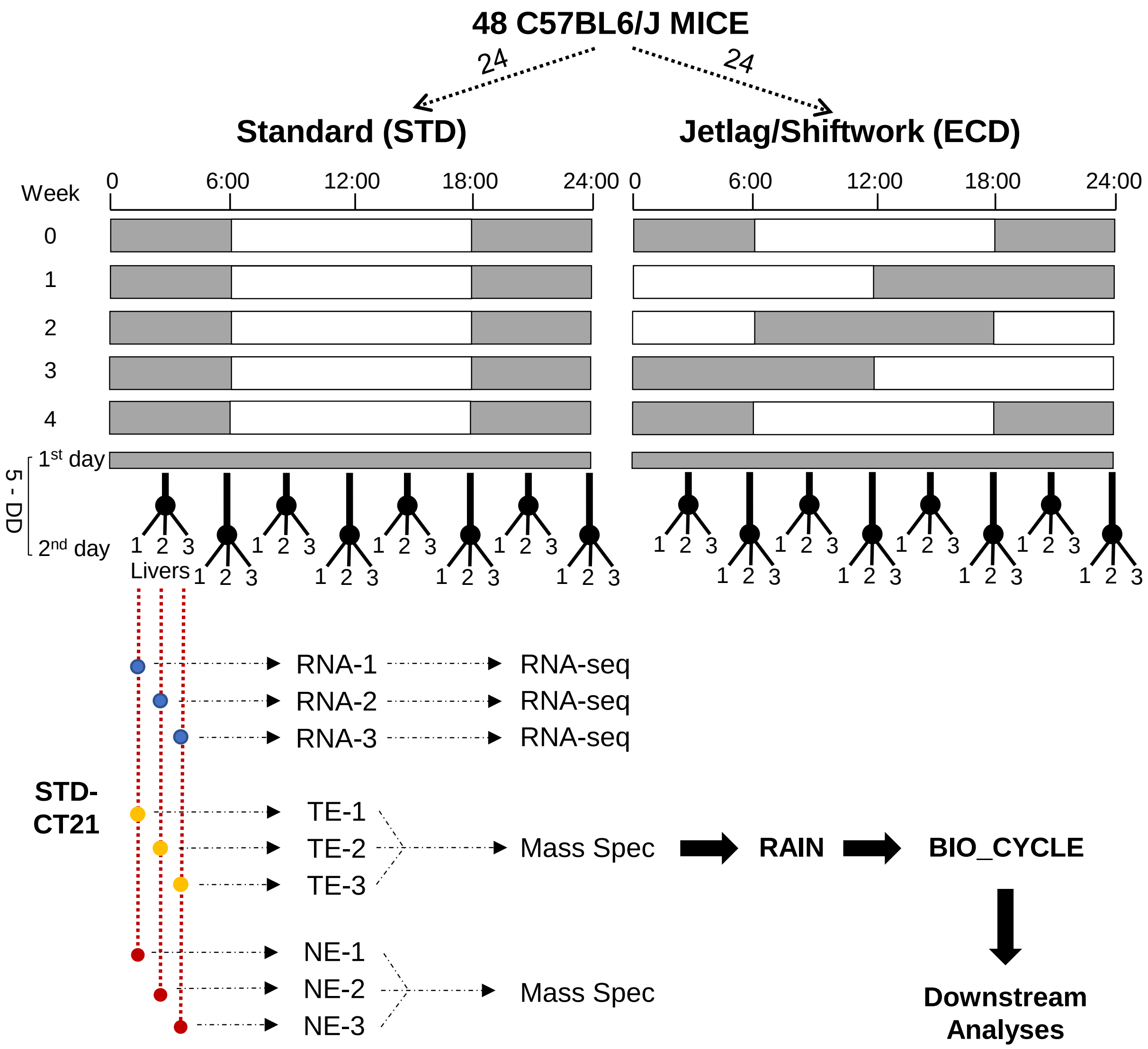

Supplementary Figure 1: Experimental paradigm. 48 C57BL6/J mice of similar ages were divided into 16 groups of 3 mice/group. 8 groups were subjected to standard light-dark cycle (STD, ON at 6am and OFF at 6pm), while the remaining 8 groups were subjected to Jetlag/Shiftwork light-dark regiment (ECD; 4 cycles of 6-hr phase-advance light shift for 1 week per cycle). After the last shift, animals were recovered under the STD cycle for 1 week. All animals were then subjected to constant darkness (DD). Tissues from each group were collected under DD at 3-hr interval starting on the 2nd day of the 5th week. Each tissue was then processed for RNA extract, total extract (TE) and nuclear extract (NE), individually. RNA-seqs were performed for each RNA extract. For mass spectrometry, equal amount of the 3 extracts from the same group and compartment were pooled before the analysis. All time series of abundance were then subjected to both RAIN and BIO_CYCLE algorithms for determination of rhythmicity before proceeding to downstream analyses.

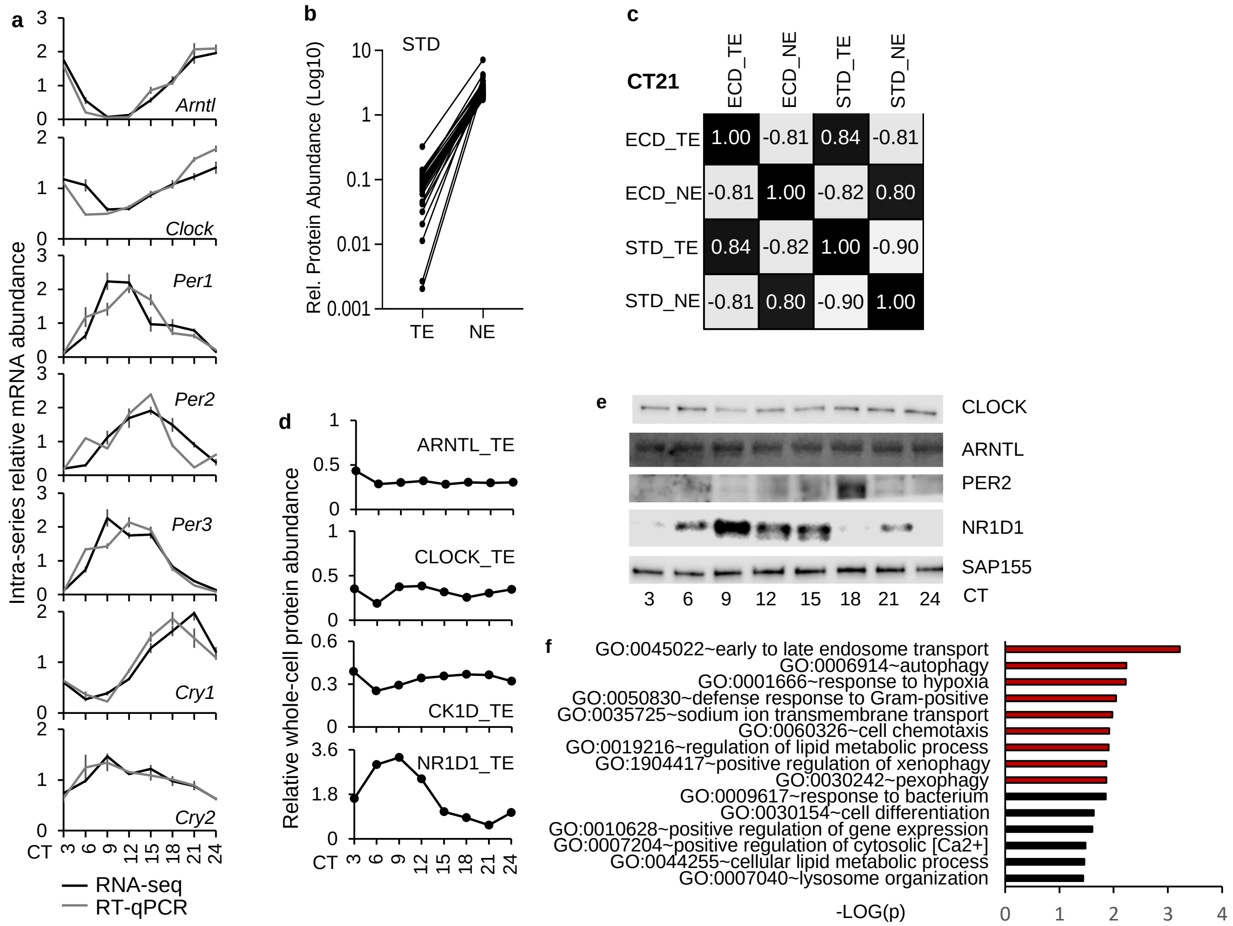

Supplementary Figure 2: STD Validations. (**a**) Comparative abundance patterns of core clock transcripts detected by RNA-seq and RT-qPCR. (**b**) Series-average relative abundance in whole-cell and nuclear extracts of some known nuclear proteins with 20+ folds of NE/TE enrichment (Log10 scale). (**c**) Pearson's correlation across compartments and conditions of all proteins quantified by mass spectrometry at CT21. (**d**) Patterns of whole-cell protein abundance of core clock components: ARNTL, CLOCK, CK1D and NR1D1 detected by mass spectrometry under STD. (**e**) Western blot of total liver extracts under STD, representative of 3 biological replicates. (**f**) G.O. enrichment analysis of the whole-cell circadian proteome under STD. Red bar – known circadian associated process.

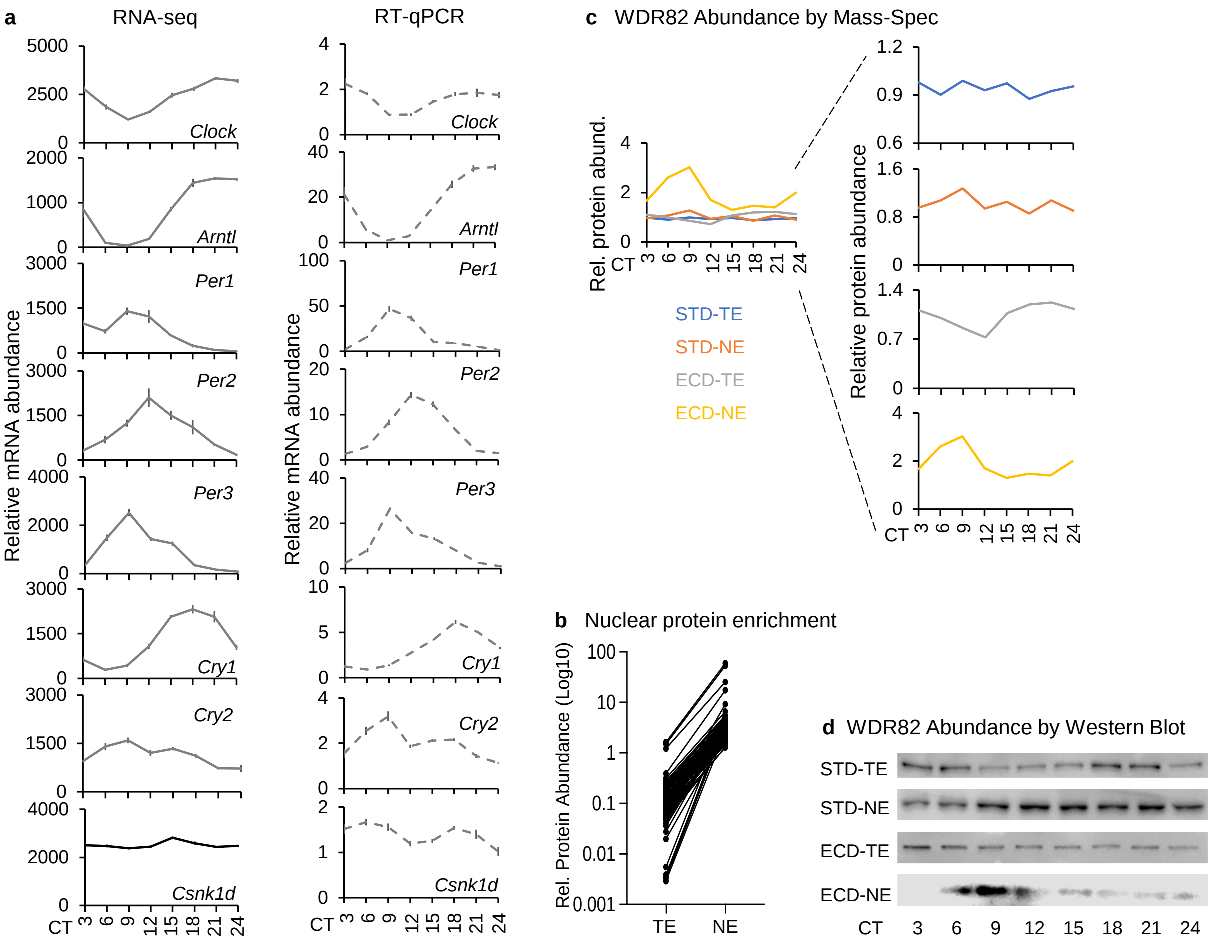

Supplementary Figure 3: ECD Validations. (**a**) Comparative abundance patterns of core clock transcripts detected by RNA-seq and RT-qPCR. (**b**) Series-average relative abundance in whole-cell and nuclear extracts of some known nuclear proteins with 20+ folds of NE/TE enrichment (Log10 scale). (**c**) Comparative patterns of WDR82 protein abundance in whole-cell or nucleus under STD or ECD detected by mass spectrometry. (**d**) Western blots of WDR82 in whole-cell or nuclear extract under STD or ECD, representative of 2+ biological replicates.

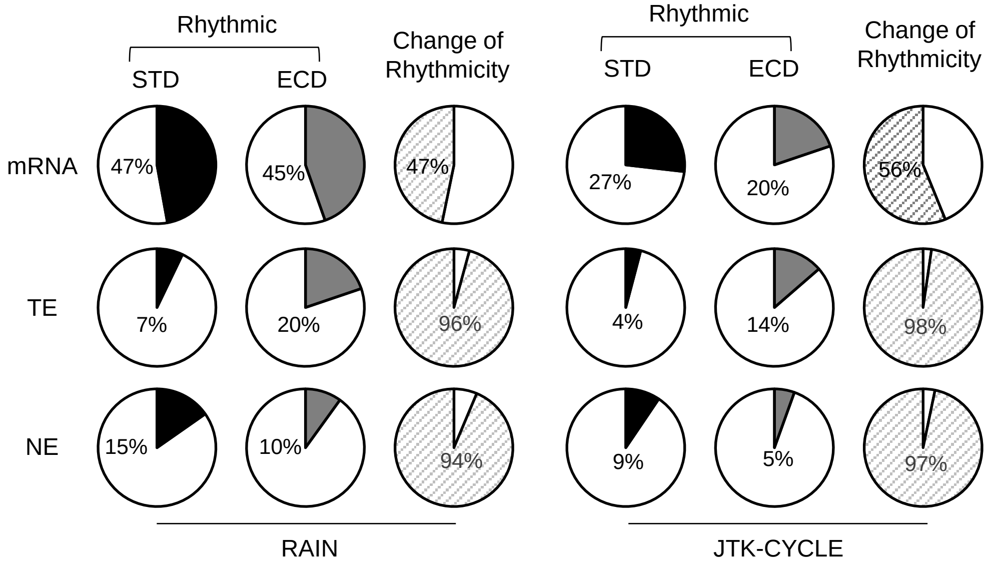

Supplementary Figure 4: Rhythmic analysis using RAIN or JTK-CYCLE algorithm as described in Figure 3

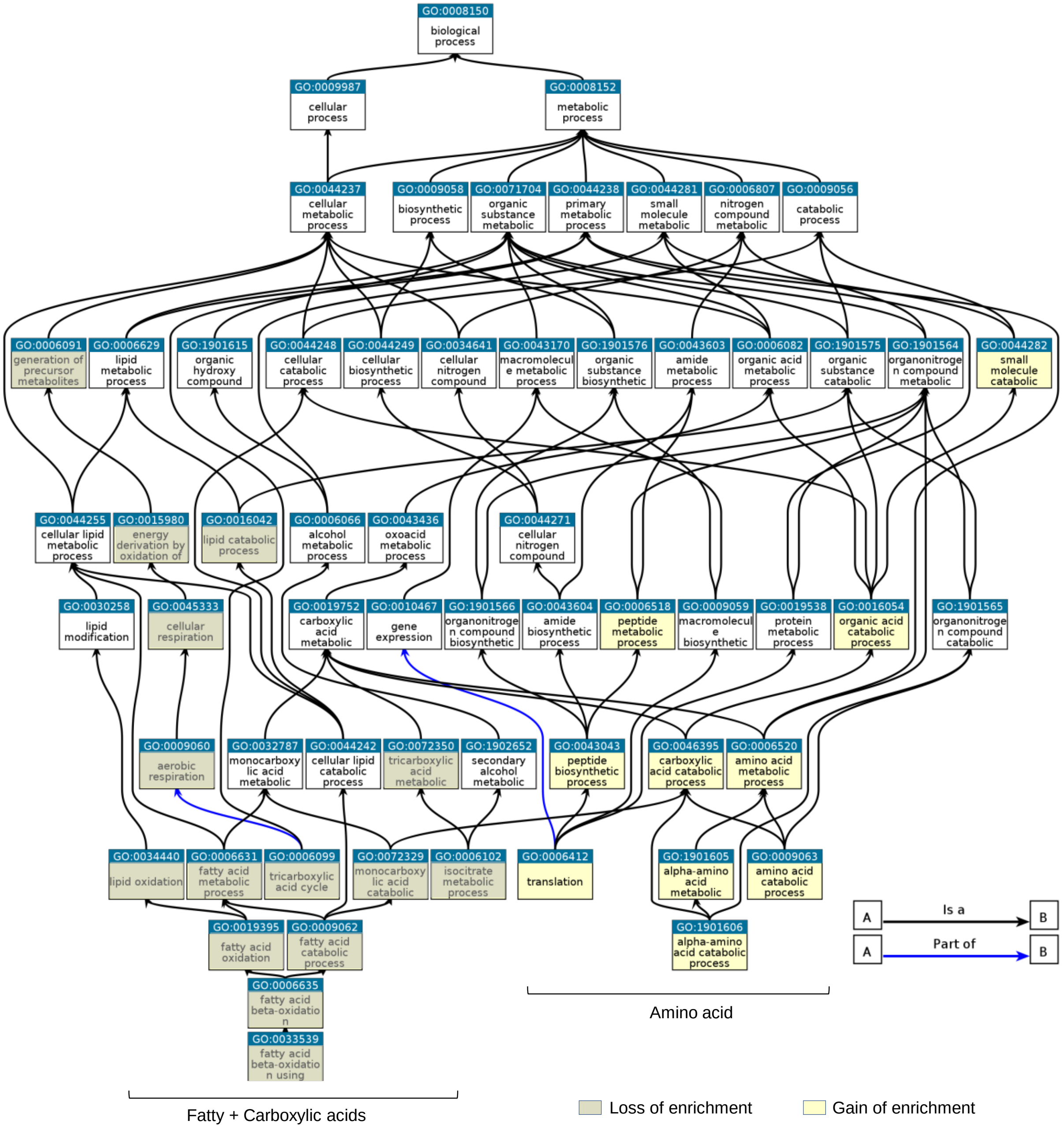

Supplementary Figure 5: G.O. map of change of enrichment of terms related to fatty acid, carboxylic acid and amino acid in response to ECD as described in Figure 6e
