# Supplementary material for: Environmental circadian disruption re-writes liver circadian proteomes
Source: Nat Commun. 2024 Jul 1;15:5537. doi: 10.1038/s41467-024-49852-3 (PMC11220080; doi:10.1038/s41467-024-49852-3)
Supplement: Supplementary file 3 — Description of Additional Supplementary Files [file 41467_2024_49852_MOESM3_ESM.pdf]

Description of Additional Supplementary Files

Supplementary Data 1: Abundant quantification of transcript, whole-cell protein and nuclear protein in the same set of male mouse liver under standard (STD) condition at 8 time point throughout a circadian cycle. Sheet 1 STD mRNA, comparative abundance of total mRNA measured by RNA sequencing. Sheet 2 STD_TE, comparative whole-cell protein abundance measured by mass spectrometry. Sheet 3 STD_NE, comparative nuclear protein abundance measured by mass spectrometry. Sheet 4 STD_22, genes of which transcript, whole-cell protein and nuclear protein are rhythmic. Sheet 5 STD_6937, genes of which transcript(s) and protein(s) are successfully quantified.

Supplementary Data 2: Comparative transcript abundance between standard (STD) and Environmental Circadian Disruption (ECD) conditions throughout a circadian cycle. Sheet 1_ECD_mRNA, comparative abundance of total mRNA measured by RNA sequencing after ECD. Sheet 2_LOR_mRNA, genes of which mRNA abundance is rhythmic under STD but not rhythmic after ECD. Sheet 3_GOR_mRNA, genes of which mRNA abundance is not rhythmic under STD but rhythmic after ECD. Sheet 4_ROR_mRNA, genes of which mRNA abundance is rhythmic under STD and after ECD.

Supplementary Data 3: Comparative protein abundance between standard (STD) and Environmental Circadian Disruption (ECD) conditions throughout a circadian cycle. Sheet 1_ECD_TE, comparative abundance of whole-cell protein measured by mass spectrometry after ECD. Sheet 2_LOR_TE, genes of which whole-cell protein abundance is rhythmic under STD but not rhythmic after ECD. Sheet 3_GOR_TE, genes of which whole-cell protein abundance is not rhythmic under STD but rhythmic after ECD. Sheet 4_ROR_TE, genes of which whole-cell protein abundance is rhythmic under STD and after ECD. Sheet 5_ECD_NE, comparative abundance of nuclear protein measured by mass spectrometry after ECD. Sheet 6_LOR_NE, genes of which nuclear protein abundance is rhythmic under STD but not rhythmic after ECD. Sheet 7_GOR_NE, genes of which nuclear protein abundance is not rhythmic under STD but rhythmic after ECD.

Sheet 8_ROR_NE, genes of which nuclear protein abundance is rhythmic under STD and after ECD.

Supplementary Data 4: 5028 genes of which transcript(s) and protein(s) are successfully quantified after ECD.

Supplementary Data 5: Proteins harboring Nuclear Localization Sequence (NLS) and/or Nuclear Export Sequence (NES). Sheet 1_NLS_STD6937RhyTE, protein members of the 6937 STD population that are rhythmic in whole cell but not rhythmic in the nucleus. Sheet 2_NLS_STD6937RhyNE, protein members of the 6937 STD population that are not rhythmic in whole cell, but rhythmic in the nucleus. Sheet 3_NLS_ECD5028RhyTE, protein members of the 5028 ECD population that are rhythmic in whole cell but not rhythmic in the nucleus. Sheet 4_NLS_ECD5028RhyNE, protein members of the 5028 ECD population that are not rhythmic in whole cell, but rhythmic in the nucleus.
